# No evidence for a pathogen associated with pulmonary MALT lymphoma: a metagenomics investigation

**DOI:** 10.1186/s13027-021-00351-w

**Published:** 2021-02-06

**Authors:** Raphaël Borie, Valérie Caro, Hilario Nunes, Marianne Kambouchner, Aurélie Cazes, Martine Antoine, Bruno Crestani, Karen Leroy, Christiane Copie-Bergman, Aurelia Kwasiborski, Christophe Hennequin, Mathias Vandenbogaert, Véronique Hourdel, Jacques Cadranel

**Affiliations:** 1 Service de Pneumologie A, Centre de référence des maladies pulmonaires rares, AP-HP, Hôpital Bichat and Université de Paris and INSERM U1152, Paris, France; 2 Environment and Infectious Risks unit, Institut Pasteur, Paris, France; 3 Service de Pneumologie, Centre de référence des maladies pulmonaires rares, AP-HP, Hôpital Avicenne, Bobigny, France; 4 Service d’Anatomie pathologique, AP-HP, Hôpital Avicenne, Bobigny, France; 5 Service d’Anatomie pathologique, AP-HP, Hôpital Bichat, Paris, France; 6 Service d’Anatomie pathologique, AP-HP, Hôpital Tenon and GRC#4 Theranoscan Sorbonne Université, Paris, France; 7 Laboratoire de Biologie et génétique moléculaire, APHP, Hôpital Cochin, Paris, France; 8 Département de Pathologie, APHP, Groupe Henri Mondor-Albert Chenevier, INSERM U955, Université Paris Est, F-94010 Creteil, France; 9 Service de Parasitologie-Mycologie, Sorbonne Université, Inserm, Centre de Recherche Saint-Antoine, CRSA, AP-HP, Hôpital Saint-Antoine, F-75012 Paris, France; 10 Service de Pneumologie et Oncologie thoracique, Centre de référence des maladies pulmonaires rares, AP-HP, Hôpital Tenon and GRC#4 Theranoscan, Sorbonne Université, Paris, France

## Abstract

Mucosa-associated lymphoid tissue (MALT) lymphoma is generally associated with chronic antigen stimulation: auto-antigens or of microbial origin. Only one study suggested association between *Achromobacter xylosoxidans* and pulmonary MALT lymphoma. We aimed to investigate the presence of virus or any infectious agents in pulmonary MALT lymphoma by using metagenomic next-generation sequencing (mNGS).

All lung samples were centrally reviewed. The t(11;18) (q21;q21) was evaluated by FISH analysis. The snap frozen large lung biopsies were analyzed by mNGS. After lung biopsies homogenization total nucleic acids (RNA and DNA) were extracted, amplified and classified according to their taxonomic assignment, after exclusion of host DNA.

We included 13 samples from pulmonary MALT lymphoma (mean age: 60.3 years, 7 women, 3 with auto-immune background) and 10 controls. The diagnosis of MALT lymphoma was confirmed for the 13 samples, 3 showed API2-MALT1 translocation (23%). No evidence of the presence of a specific pathogen was clearly identified in the group of patients with pulmonary MALT lymphoma. We identified*A. xylosoxidans* sequence in 4/13 patients and in 4/10 controls.

This study did not find evidence for a DNA or RNA virus, a fungi, a parasite or a bacteria associated with pulmonary MALT lymphoma either in the stroma or in tumor cells.

Though rare, mucosa-associated lymphoid tissue (MALT) lymphoma is the most common pulmonary B-cell lymphoma. The disease is slow-growing with an asymptomatic chronic alveolar opacity visible on radiography [1]. Interestingly MALT lymphomas are generally associated with chronic antigen stimulation, regardless of whether the antigens are auto-antigens or of microbial origin [2, 3]. *Helicobacter pylori* was initially detected in almost 90% but only 65-70% in recent series of gastric biopsies from patients with gastric MALT lymphoma, and the lymphoma is usually cured by antibiotics [4]. Other infectious agents have been suggested as possible trigger of MALT lymphoma at other sites though with less evidences [5, 6]. A causal relationship has been suggested between *Campylobacter jejuni*infection and small intestine MALT lymphoma as well as between hepatitis C virus infection and some cases of splenic MZ lymphoma [2]. Several studies have found an association between *Borrelia burgdorferi* infection and skin MALT lymphoma, and between *Chlamydophila psittaci *and ocular adnexal MALT lymphoma [7, 8].

Only one study suggested an infectious cause of pulmonary MALT lymphoma. Using a 16S RNA-based approach, the authors found DNA from *Achromobacter xylosoxidans* in 57/124 pulmonary MALT lymphomas *vs*15/82 controls (*p *= 0.004) [9]. Chronic auto-antigen stimulation may also be a contributing factor, and a 5-fold increased risk of extranodal lymphoma has been observed in Sjögren’s syndrome [10]. Finally, diverse oncogenic alterations have been detected in MALT lymphoma [11], and the t(11;18) (q21;q21) translocation has been associated with resistance to *H. pylori*-targeting antibiotics [12]. t(11;18) is the most frequently found, detected in 42% of pulmonary, 22% of gastric, and 15% of intestinal, though absent in most cases of salivary gland, and liver MALT lymphoma [13].

By using an agnostic metagenomic next-generation sequencing (mNGS) approach, we aimed to investigate without prior hypothesis the presence of infectious agents sequences associated with pulmonary MALT lymphoma, in conjunction with the presence of oncogenic alteration and auto-immune disease [14].

From a retrospective cohort of 63 patients with a histological diagnosis of pulmonary MALT lymphoma from 1993 to 2008, monitored in three French hospitals, we enrolled 13 patients (mean age: 60.3 years, 7 women, 5 active or past smokers, 3 with auto-immune background) with surgical biopsy to insure sufficient available amount of snap frozen lung tissue specimens including tumor cells and stroma for mNGS exploration [1]. Every patient had a localized disease assessed by clinical examination and thorax and abdominal CT, 10/13 patients had a systematic gastric endoscopy, 8/13 a bone marrow biopsy and 7/13 a 18F- FDG PET.Two patients had a Sjögren syndrome and one a systemic lupus erythematosus. Normal lung tissue specimens obtained from 10 patients (mean age: 60.3 years, 3 women, 8 active or former smokers, none with auto-immune background) undergoing lung surgery for removal of a primary lung tumor served as controls. This study was approved by the local ethics committee (CPP Ile de France 1, no. 0811760). All samples were centrally reviewed and classified according to the latest WHO classification of lymphoid neoplasms by a core of experienced pathologists (MK, AC, MA, CCB) [3]. The t(11;18) (q21;q21) was evaluated by FISH analysis on 3 μm paraffin embedded tissue sections using breakapart FISH DNA probes for MALT1 (Dako A/S, Glostrup, Denmark) and fusion probes for API2-MALT1 et IGH-MALT1 (Abbott Vysis, Chicago, USA) according to the manufacturer's recommendations. Slides were analyzed with a Zeiss Axioplan2 fluorescence microscope equipped with microscope-specific double filters (XF53, Omega Optical, Brattleboro, VT) suitable for the fluorescein isothiocyanate and Texas Red labeled split-signal probes. Slides were analyzed by an experienced pathologist (CCB) with a 100× oil immersion objective. Scoring of the hybridization signals using breakapart probes was performed according to the algorithm published by Haralambieva *et al.*[15]. Using this algorithm, the cutoff value established in negative control tissues consisting of reactive lymphoid tissue samples is 10%.

The snap frozen lung biopsies provided from pulmonary MALT lymphoma patients and control patients were subjected to mNGS. After lung biopsies homogenization and depletion of host DNA, total nucleic acids were extracted using TRIzol Reagent (Invitrogen) according to manufacturer´s recommendations except for DNA purification step, which was performed with QiaAmp DNA mini kit (Qiagen). RNA and DNA amplification were performed using QuantiTect Whole Transcriptome Kit and REPLI-g kit (Qiagen) respectively. All amplified nucleic acids were pooled in equal concentrations to prepare NGS libraries with TruSeq DNA PCR-free Library preparation kit. The run was performed on HiSeq 2500 sequencer (Illumina) resulting in 2 x 150 nucleotides paired-end reads. All raw data were first cleaned by trimming adapters, by removing low quality sequence reads, duplicates and host contaminant residues. The filtered reads were classified according to their taxonomic assignment with Kaiju (v. 1.4.1) and then *de novo* assembled with CLC (v. 9.5.3). Generated contigs and unassembled reads were then aligned with BLAST against a reference database (Genbank, nr) to refine taxonomic classification results. Raw data were deposited in the NCBI Sequence Read Archive under bioproject ID PRJNA664596.

All 13 MALT-lymphoma samples showed dense proliferation of small CD20+ lymphocytes infiltrating the lung. Characteristic lymphoepithelial lesions were observed in 9/13 cases (69%). Large cells were absent in all samples. Plasma cells were observed in 3/13 cases (23%). Staining for CD5, CD10, CD23, IgD, cyclin D1 and CD30 were always negative in tumoral cells. Expression of BCL2 was positive in the nine cases for which it was performed. The clonality of B-cells was available for five patients and always positive. All samples were tested for the presence of API2-MALT1 translocation by FISH, and 3 were positive (23%). None of the patients with auto-immune background presented an API2-MALT1 translocation.

The microbial diversity in the patients and controls lungs revealed by mNGS analysis was depicted by the global taxonomic classification of sequenced reads (Fig. 1). No evidence of the presence of a specific pathogen was clearly identified in the group of patients with pulmonary MALT lymphoma. Actually, we observed some prominent bacterial genera as previously described in lung tissue microbiota [16] and no significant difference in the microbial profile could be established between the two groups. Of note, we have identified in 4/13 patients and in 4/10 controls the presence of *A. xylosoxidans* sequence. These results did not support a significant involvement of *A. xylosoxidans* in the pathogenesis of this MALT lymphoma in this series [9]. In addition, we didn’t identify any sequences of any mycobacteria nor *Chlamydia psittaci*, although previous results reported its possible involvement in non-gastrointestinal MALT lymphomas [7, 8]. Remarkably, one pulmonary MALT patient was significantly positive for the presence for *Pneumocystis jirovecii*. Although a huge number of specific reads and contigs were retrieved, we could not confirm the result by specific PCR.

**Fig. 1:**
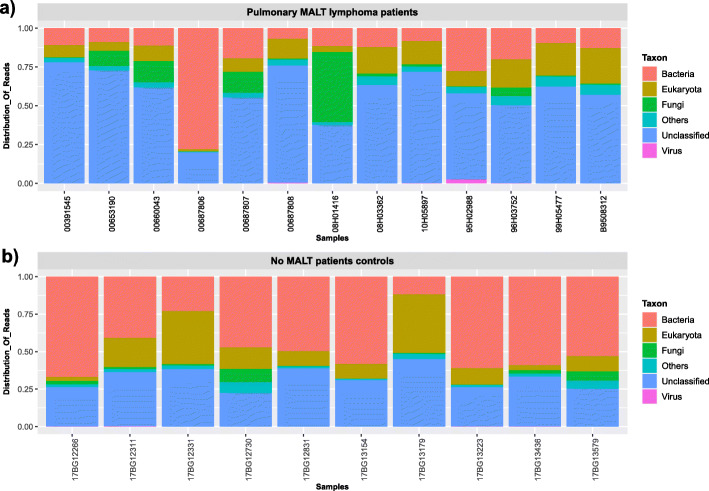
Taxonomic profiles of **a**) lungs from 13 pulmonary MALT lymphoma patients and **b**) lungs from 10 control patients. Filtered metagenomics NGS reads were classified by using Kaiju software (v. 1.4.1)

This study did not find evidence for a pathogen associated with pulmonary MALT lymphoma. Previous study evidenced an increased prevalence of *A. xylosoxidans *or *Chlamydiae *[8, 9]. We cannot exclude that *A. xylosoxidans* could eventually participate in the occurrence of MALT lymphoma in an unidentified subgroup of patients. While previous studies used marker gene sequencing, such as 16S rRNA, a targeted sequencing assay limited by the breadth of detection, our study is the first looking for an agnostic metagenomic NGS approach to explore a potential link between pulmonary MALT lymphoma and all microbial agents i.e. bacteria, viruses, fungi and parasites. Actually, our study did not detect any evidence for a specific pathogen associated with pulmonary MALT lymphoma. The small number of specimens tested and the auto-immune background profile of some patients are a limitation of our study. The detection of *Pneumocystis *DNA without the demonstration of living stages of pathogen in the tissue was observed in only one patient but not in all other tissue specimens tested from MALT-lymphoma patients and controls. Considering the high prevalence of *Pneumocystis *colonization, detected in almost 25% of patient with chronic lung disease by PCR [17], we did not incriminate *Pneumocystis *in pulmonary MALT lymphoma.

In conclusion, we were not able to identify a pathogen as a causative factor of pulmonary MALT lymphoma.

## Data Availability

Raw data were deposited in the NCBI Sequence Read Archive under bioproject ID PRJNA664596.
